# Renal PET-imaging with ^11^C-metformin in a transgenic mouse model for chronic kidney disease

**DOI:** 10.1186/s13550-016-0211-x

**Published:** 2016-06-23

**Authors:** Lea Pedersen, Jonas Brorson Jensen, Lise Wogensen, Ole Lajord Munk, Niels Jessen, Jørgen Frøkiær, Steen Jakobsen

**Affiliations:** Research Laboratory for Biochemical Pathology, Institute of Clinical Medicine, Aarhus University, Aarhus, Denmark; Department of Nuclear Medicine & PET Center, Aarhus University Hospital, DK-8000 Aarhus C, Denmark

**Keywords:** PET, ^11^C-metformin, Chronic kidney disease, Mouse model

## Abstract

**Background:**

Organic cation transporters (OCTs) in the renal proximal tubule are important for the excretion of both exo- and endogenous compounds, and chronic kidney disease (CKD) alter the expression of OCT. Metformin is a well-known substrate for OCT, and recently, we demonstrated that positron emission tomography (PET) with 11C-labelled metformin (^11^C-metformin) is a promising approach to evaluate the function of OCT. The aim of this study is therefore to examine renal pharmacokinetics of ^11^C-metformin and expression of OCTs in a transgenic (RenTGF-β1) mouse model of CKD.

**Methods:**

Age- and sex-matched RenTGF-β1 (Tg) and wildtype (WT) mice were used (5–8/group). Animals received an iv bolus of ^11^C-metformin followed by 90-min dynamic PET and MRI scan. PET data were analysed using a one-tissue compartment model. Renal protein abundance of OCT2 (by Western blot) as well as OCT1, OCT2, and MATE1 messenger RNA (mRNA) (by RT-PCR) was examined.

**Results:**

Protein expression of the basolateral uptake transporter OCT2 was 1.5-fold lower in Tg mice compared to WT mice while OCT1 and MATE1 mRNA expression did not differ between the two groups. The influx rate constant of ^11^C-metformin in renal cortex (*K*_1_) was 2.2-fold lower in transgenic mice whereas the backflux rate constant (*k*_2_) was similar in the two groups, consistent with protein expression. Total body clearance (TBC) correlated within each group linearly with *K*_1_.

**Conclusions:**

In conclusion, this study demonstrates that both renal OCT2 expression and ^11^C-metformin uptake are reduced in CKD mice. This potentially makes ^11^C-metformin valuable as a PET probe to evaluate kidney function.

## Background

The prevalence of chronic kidney disease (CKD) is dramatically increasing worldwide [1–5]. Renal accumulation and abnormal regulation of extracellular matrix molecules are both characteristics of CKD and can be directly linked to impaired kidney function. Importantly, diabetic nephropathy is the most frequent cause of end-stage renal disease accounting for more than half of new diagnoses [6].

Both acute and chronic kidney failure are associated with altered expression of numerous transport proteins along the nephron including organic cation transporters (OCTs) located to the proximal tubules [7, 8]. OCTs are responsible for renal excretion of both exo- and endogenous organic cations in the kidneys, and decreased expression may lead to the accumulation of these compounds to toxic levels [9, 10]. The anti-diabetic drug metformin is a well-known substrate for OCTs, and 11C-labelled metformin (^11^C-metformin) was recently demonstrated as a PET probe for the evaluation of renal OCTs in rats and pigs [11]. In proximal tubule of mice, OCT2 (and to a lesser degree OCT1) mediates basolateral uptake of metformin whereas the multidrug and toxin extrusion protein 1 (MATE1) is responsible for excretion into the tubule lumen in a H^+^-coupled electroneutral manner [12, 13].

CKD can be studied in a mouse model with TGFβ-induced chronic kidney disease (RenTGF-β1). These mice express proximal tubular dysfunction, tubular basement membrane thickening, interstitial fibrosis, and proteinuria when they are approximately 4-month old [14, 15]. The structural changes are mainly located to the basolateral part of the cells along the nephron which could lead to altered OCT2 function.

Presently, it is unknown whether ^11^C-metformin can be used as a renal in vivo imaging tool to detect functional abnormalities of OCTs in CKD. Therefore, the present study was designed to investigate renal pharmacokinetics of ^11^C-metformin and the expression of OCTs in a mouse model for CKD.

## Methods

### Mouse model

For the kinetic modelling study, we used the previously described RenTGF-β1 transgenic (Tg) mouse strain expressing mutated porcine TGF-β1 under control of the Ren-1^c^ promotor [16]. Tg females (*n* = 8) between 4- and 5-month old were used, and age- and sex-matched wildtype (WT) littermates (*n* = 5) were used as control animals. To further investigate the relative importance of OCT1/2 and MATE1, we calculated total body clearance (TBC) for animals used in [17]. This included controls at baseline and after treatment with cimetidine (an OCT1/2 inhibitor) or pyrimethamine (a MATE1 inhibitor) and OCT1/2 knock-out mice. All animals were housed at the animal facility at the Aarhus University and handled according to the guidelines and procedures approved by the Animal Experiments Inspectorate, Denmark. Special characteristics for the mice models have been published previously [15, 18, 19].

### mRNA expression by real-time RT-PCR, electrophoresis, and immunoblotting analysis

From Tg and WT mice, messenger RNA (mRNA) was extracted as previously described (*n* = 8/group) and amounts of OCT1, OCT2, and MATE1 were measured [14]. The primer pairs used in the study were murine OCT1: forward 5′-CATCTTGTACCAGGTGGCCT-3′, reverse 5′-CCGCCTGAGTGGTTCTCTTC-3′; murine OCT2: forward 5′-AAATGGTCTGCCTGG TCAAC-3′, reverse 5′-AGGCCAACCACAGCAAATAC-3′; murine MATE1: forward 5′-CTGCTCTTCAGACAGGACCC-3′, reverse 5′-TGACAAGGTTAG CTGCGATG-3′; and GAPDH: forward 5′-ATGTTCCAGTATGACTCCACTCACG-3′, reverse 5′-GAAGA CACCAGTAGACTCCACGACA-3′. Proteins from renal tissue (*n* = 7/group) were analysed with Western blotting as previously described [20] with OCT2 rabbit polyclonal antibody (1:500, LS-C80615, LifeSpan Bioscience, Seattle, WA, USA).

### ^11^C synthesis and microPET/MR

^11^C-metformin was prepared as previously described [11]. Dynamic PET- and anatomical MR-imaging were conducted in Tg (*n* = 8) and WT (*n* = 5) mice using Mediso nanoScan PET/MR (Mediso Ltd., Hungary). Anaesthesia was induced in a chamber filled with 4–5 %/0.5 % isoflurane/oxygen inhalation mixture. A catheter was placed into the tail vein, and sterile saline (~0.2 mL) was administered to insure adequate hydration. A single bolus of ^11^C-metformin (7.7 ± 4.0 MBq/mouse) was injected via the catheter followed by 90-min dynamic PET- and anatomical MR-imaging. Body temperature was kept at 36–37 °C throughout the intervention, and respiration frequency was monitored. Dynamic PET data were reconstructed as previously reported [17].

### PET image analysis

Multiple regions of interest (ROIs) were placed on the renal cortex, heart, and liver using PMOD version 3.5 (PMOD Technologies Ltd, Zurich, Switzerland) creating a volume of interest (VOI). Half-moon shaped renal cortex VOIs were defined on an average image of the 33 PET frames. Subsequently, it was verified that the VOIs were placed within the renal cortex in each frame. Hepatic VOIs were drawn on the first 25 frames where it can easily be identified and averaged. The heart was used as an image-derived input function. Frames from the first 20 s were averaged and ROI circles with a diameter of 15 pixels were placed on the six most intensive slices in the middle of the heart. Correct positioning of the liver and heart VOIs were checked in each time frame and adjusted if needed. Time-activity curves (TACs) were generated from the VOIs.

### Kinetic modelling

Dynamic PET data for each kidney cortex were fitted using a one-tissue compartment model with two parameters: *K*_1_, the influx rate constant (from blood into the tissue compartment) and *k*_2_, the backflux rate constant (out of the tissue compartment). The kinetic parameters were estimated by minimizing the residual sum of squares (RSS) using the Levenberg-Marquard algorithm. A single mouse was excluded from the analyses because the model fit yielded non-physiological parameter estimates.

TBC during 90 min was calculated based on the imaged-derived input function from the following equation:

$$ \mathrm{T}\mathrm{B}\mathrm{C}=\frac{\mathrm{ID}\ }{{\mathrm{AUC}}_{\left(0-90\  \min \right)}} $$

where ID is injected dose relative to kilogram body weight and AUC_(0–90 min)_ represents Area Under the Curve of the image-derived input function up to 90 min.

### Statistics

All data are presented as mean ± standard error of mean (SEM), and the statistical analyses were performed using Sigma Plot version 11.0. The Kolmogorov-Smirnov test with Lillefors’ correction was used to test all data for normality, and the unpaired data were compared using Student’s *t* test. Data that were not normally distributed were compared using the Mann-Whitney rank sum test. Differences between groups were considered significant when *P* < 0.05.

## Results

### Reduced mRNA and protein expression of renal OCT2 in Tg mice

**Fig. 1:**
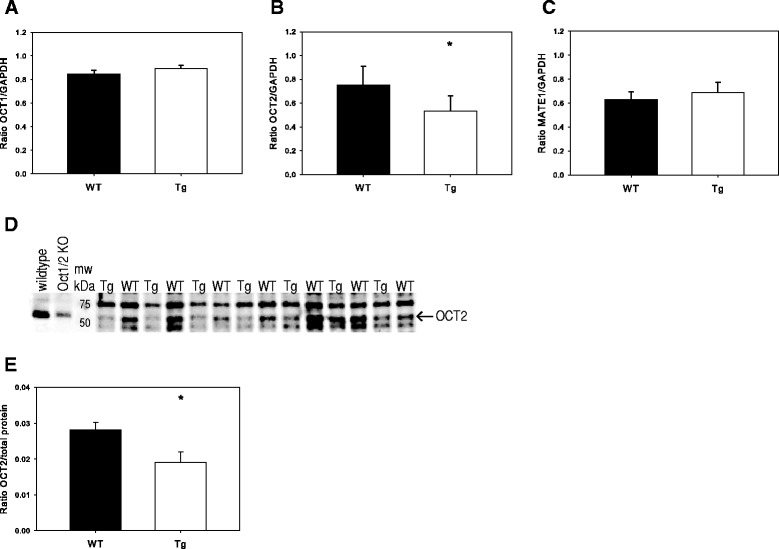
Expression of OCT2 is reduced in CKD mice. **a**–**c** Whole kidney tissue expression of **a** OCT1 mRNA, **b** OCT2 mRNA, and **c** MATE1 mRNA (values are mean ± SEM, *n* = 8) (*P = 0.015). **d**–**e** Western blot for OCT2 protein (**d**) with a wildtype mouse as positive control (lane 1) and a OCT1/2 KO mouse as negative control (lane 2). The band (*arrow*) corresponding to the positive control in the Tg and WT mice is assumed to represent OCT2 protein abundance (**e**) (values are relative to total protein and represent mean ± SEM, *n* = 7) (*P = 0.026)

The mRNA expression of different relevant transport proteins was investigated in total kidney tissue from a different group of WT and Tg mice (*n* = 8/group). The expression of OCT1 was not significantly different between WT and Tg mice (Fig. 1a); however, OCT2 mRNA was 1.4-fold lower in Tg versus WT mice (*P* = 0.015, Fig. 1b). MATE1 expression did not differ significantly between WT and Tg mice (Fig. 1c). Protein levels of OCT2 were 1.5-fold lower in Tg mice when investigated with Western blotting in membrane fraction of kidney tissue (*n* = 7/group, *P* = 0.026, Fig. 1d, e).

### Rapid renal clearance of ^11^C-metformin visualised by PET

**Fig. 2:**
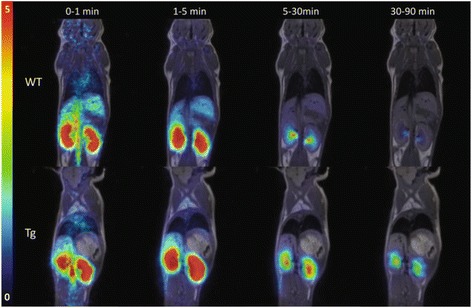
Coronal whole body PET with ^11^C-metformin merged with T1-weighted MRI-sequence in a WT mouse (*upper panel*) and a Tg mouse (*lower panel*). The projection is posterior to the liver and heart. Radioactivity in the kidneys peaks from 1 to 5 min and decreases towards 90 min because of extensive urinary excretion. *Scale bar to the left* represents standard uptake value (SUV) 0–5

**Fig. 3:**
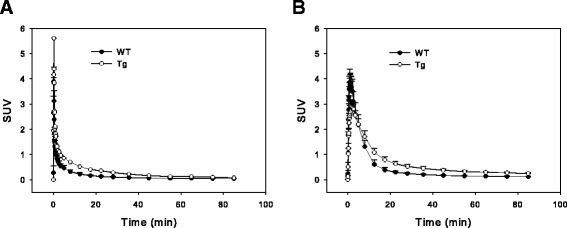
Time-activity curves of ^11^C-metformin in the image-derived input function (**a**) and in the kidneys (**b**) in Tg and WT mice. Data are expressed as standard uptake value (SUV) = concentration [kBq/mL] × (body weight [g]/injected dose [kBq]). The data represent means + SEM. *Closed circles* = WT; *Open circles* = Tg

After 90-min dynamic PET-imaging, the highest amount of radioactivity was found in the urinary bladder. The renal distribution of ^11^C-metformin peaked from 1–5 min and decreased towards 90 min on the co-registered PET/MR image due to extensive urinary excretion (Fig. 2). Time-activity curves for WT and Tg mice were generated (Fig. 3). Of note is the absence of pronounced visual differences in ^11^C-metformin kinetics between WT and Tg mice during the first 30 min.

### Impaired total body clearance of ^11^C-metformin correlates with decreased uptake in renal cortex of Tg mice

**Fig. 4:**
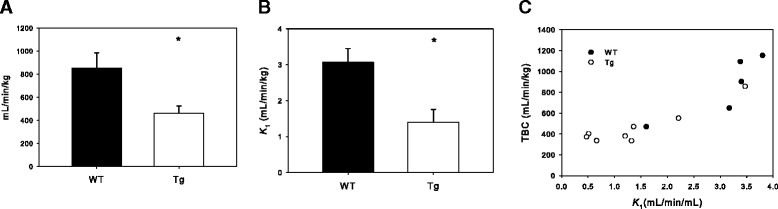
Kinetic analysis of dynamic PET data. **a** TBC of ^11^C-metformin in WT (*n* = 5) and Tg (*n* = 8) mice determined from image-derived input function. **b** *K*_1_ in renal cortex determined from compartmental analysis. **c** Correlation between *K*_1_ and TBC (*P < 0.05). *Closed circles* = WT; *Open circles* = Tg

TBC of ^11^C-metformin was determined using the image-derived input function and was 1.8-fold lower in Tg compared to WT mice (*P* = 0.010, Fig. 4a and Table 1). No difference was observed between the left and right kidney; consequently, rate constants were expressed as means of both kidneys. The influx rate constant in renal cortex (*K*_1_) was 2.2-fold lower in Tg mice (*P* = 0.027, Fig. 4b). *k*_2_ did not differ significantly between the two groups (*P* = 0.11, data not shown). TBC correlated within each group with *K*_1_ (*R* = 0.79 WT, *R* = 0.91 Tg, Fig. 4c). Logan plot of ^11^C-metformin from 20–90 min showed that the liver volume of distribution did not differ significantly between the two groups (data not shown).

**Table 1:** TBC of ^11^C-metformin in RenTGF-β1 mice (Tg), OCT1/2 KO mice, or wildtype mice treated with an OCT1/2 inhibitor (cimetidine) or MATE1 inhibitor (pyrimethamine), respectively, relative to the appropriate control mice

Group	Fold-change in TBC	*P* value
Control	1	N.A.
Tg (*n* = 8)	1.8 ↓	0.011
OCT1/2 KO (*n* = 4)	3.0 ↓	<0.001
Cimetidine (*n* = 5)	2.5 ↓	0.011
Pyrimethamine (*n* = 4)	1.1 ↑	0.357

*N.A.* not available

### OCT1/2 status is crucial for ^11^C-metformin TBC

Ablation of OCT1/2 or pre-treatment with cimetidine had a profound lowering effect on TBC whereas inhibition of MATE1 by pyrimethamine slightly increased TBC albeit insignificantly (Table 1).

## Discussion

Here we provide data from dynamic PET-imaging with ^11^C-metformin in a well-established mouse model of CKD. For the first time, we demonstrate that influx rate constant of ^11^C-metformin into the renal cortex is lower in a state of chronic kidney disease. Furthermore, expression of the basolateral uptake transporter OCT2 is lower in the transgenic mouse model. This finding may have implications for developing radiolabelled metformin PET-imaging as a method to evaluate kidney function changes.

In Tg mice, the structural changes and associated changes in the biochemical environment are mainly located to the basolateral part of the epithelial cells along the nephron. This is consistent with the observations that OCT2 mRNA expression levels in Tg mice are reduced compared with WT mice whereas there are no difference in the mRNA expression of the apical MATE1 transporter. The absence of changes in OCT1 mRNA level is consistent with previous findings in a rat model of chronic kidney failure [7]. To confirm the importance of the mRNA findings, we investigated protein levels of OCT2 and found a comparable reduction in the OCT2 protein expression in Tg mice. This could suggest that OCT2 expression is more sensitive to renal damage than OCT1.

Metformin is described to be a superior substrate for OCT2 than OCT1, and the capacity to transport metformin is 10–100-fold greater for OCT2 when compared to OCT1 [21]. This would make metformin excretion more sensitive to alterations in OCT2- rather than OCT1 expression, and thus, ^11^C-metformin PET is potentially an in vivo imaging probe for OCT2 evaluation.

Renal excretion of metformin in the proximal tubule accounts for approximately 80 % of renal clearance of the drug under normal conditions [12, 13, 22]. In mice, OCT1 and -2 are responsible for basolateral uptake of metformin and TBC was reported to be 4.5-fold lower in OCT1/2 knock-out mice [12]. In the present study, we found that TBC of ^11^C-metformin was 1.8-fold lower in Tg mice than WT mice and still lower after blockade or removal of OCT1/2. Thus, these findings suggest a lower expression of OCT2 in Tg mice to be responsible for the reduced TBC of ^11^C-metformin. In support of this view, the pharmacokinetic analyses of ^11^C-metformin in renal cortex revealed a lower *K*_1_ in Tg mice, i.e., the basolateral cellular uptake in renal cortex is slower as there is lower expression level of the basolateral uptake transporter. Besides the transporter mediated cellular uptake in renal cortical compartment, GFR and renal blood flow also influence *K*_1_. GFR is reported to be approximately twofold lower in 3-month-old Tg mice determined by ^51^Cr-EDTA [15], but as glomerular filtration only accounts for ~20 % of renal metformin clearance, this cannot solely explain the observed difference in *K*_1_.

MATE1 is mainly responsible for the excretion of metformin into proximal tubule lumen, and TBC of metformin has been shown to be approximately fourfold lower in MATE1^−/−^ mice [13]. Interestingly, the blockade of MATE1 by pre-treatment with pyrimethamine did not lower TBC of ^11^C-metformin perhaps reflecting higher kidney versus liver abundance of MATE1. We found no difference in backflux rate constant (*k*_2_) of ^11^C-metformin between the two groups consistent with comparable MATE1 expression levels. Overall, OCT2 seems to be responsible for a lower ^11^C-metformin uptake in the renal cortex in our mouse model for CKD.

In clinical practice, metformin treatment is only recommended for individuals without CKD or mild-to-moderate stages of CKD due to the risk of lactic acidosis, which is a rare but potentially life-threatening complication [23, 24]. With no clear-cut correlation between plasma levels of metformin and prevalence of lactic acidosis, the literature is currently conflicting about the safety of the drug and national restrictions are excluding a number of patients from the beneficial effects of metformin. Here we present a new approach to understand the mechanisms by which metformin is handled by the kidneys in stages of CKD. This approach could form the basis of future clinical studies and ultimately differentiate between patients with CKD who will tolerate metformin treatment and who will not.

Possible limitations of this study relates to quantification in small-animal PET. TBC and kinetic modelling were based on the image-based input function from the heart rather than an input function measured by blood sampling. Fast and repeated blood sampling from mice is troublesome partly because of the non-triviality of obtaining a proper arterial line and partly because of the limited amount of blood that can be safely taken. The heart region is assumed to be proportional to plasma concentration of ^11^C-metformin due to negligible uptake into erythrocytes during the scan period (Lars Chr. Gormsen, submitted). However, this proportionality can be affected by the size of the heart and haematocrit. The heart size tends to be smaller in Tg mice when evaluated on PET images. This would include more cardiac tissue with minimal ^11^C-metformin uptake in the region and thereby dilute the concentration of ^11^C-metformin in Tg mice. Haematocrit is also lower in Tg mice [18] but would account for the opposite effect because of negligible uptake of ^11^C-metformin into erythrocytes. The one-tissue compartment model used in the present study produced the best fit which is consistent with findings in pigs [11]. Even so, a more advanced kinetic model might enable the discrimination of some of the different components (blood flow, GFR, OCT-mediated transport, and urine flow) in renal handling of ^11^C-metformin; however, we are not able to implement such an approach on the current animal data because of limited spatial resolution inherent in small-animal microPET studies. We anticipate that some of these aspects are manageable or even non-existent in future human scans.

## Conclusions

It has previously been shown that OCT expression is affected by CKD. In this study, we show that diminished OCT2 expression in a mouse model of CDK reduced the renal uptake of ^11^C-metformin and that TBC of ^11^C-metformin is lowered profoundly after ablation of OCT1/2 or pre-treatment with cimetidine. Thus, ^11^C-metformin PET warrants further investigation to assess OCT expression and renal function in vivo.

## Abbreviations

AUC, area under the curve; CKD, chronic kidney disease; GFR, glomerular filtration rate; ID, injected dose; MATE, multidrug and toxin extrusion protein; MRI, magnetic resonance imaging; OCT, organic cation transporters; PET, positron emission tomography; ROI, region of interest; RSS, residual sum of squares; SEM, standard error of mean; SUV, standard uptake value; TAC, time-activity curve; TBC, total body clearance; Tg, transgenic mice; VOI, volume of interest; WT, wildtype mice
